# The Impact of HBV Quasispecies Features on Immune Status in HBsAg+/HBsAb+ Patients With HBV Genotype C Using Next-Generation Sequencing

**DOI:** 10.3389/fimmu.2021.775461

**Published:** 2021-11-25

**Authors:** Ying Wang, Xiao Xiao, Shipeng Chen, Chenjun Huang, Jun Zhou, Erhei Dai, Ya Li, Lijuan Liu, Xianzhang Huang, Zhiyuan Gao, Chuanyong Wu, Meng Fang, Chunfang Gao

**Affiliations:** ^1^ Department of Laboratory Medicine, Shanghai Eastern Hepatobiliary Surgery Hospital, Shanghai, China; ^2^ Clinical Laboratory Medicine Center, Yueyang Hospital of Integrated Traditional Chinese and Western Medicine, Shanghai University of Traditional Chinese Medicine, Shanghai, China; ^3^ Department of Laboratory Medicine, The Fifth Hospital of Shijiazhuang, Hebei Medical University, Shijiazhuang, China; ^4^ Department of Laboratory Medicine, The First Affiliated Hospital of Kunming Medical University, Kunming, China; ^5^ Department of Laboratory Medicine, Mengchao Hepatobiliary Hospital of Fujian Medical University, Fuzhou, China; ^6^ Department of Laboratory Medicine, The Second Affiliated Hospital of Guangzhou University of Chinese Medicine, Guangzhou, China

**Keywords:** next-generation sequencing (NGS), hepatitis B virus (HBV), quasispecies, hepatitis B surface antigen (HBsAg), hepatitis B surface antibody (HBsAb)

## Abstract

**Background:**

This study aimed to explore the molecular mechanism of the coexistence of hepatitis B surface antigen (HBsAg) and hepatitis B surface antibody (HBsAb) serological pattern *via* intensive characterization of HBV s gene in both chronic hepatitis B (CHB) and hepatocellular carcinoma (HCC) patients.

**Method:**

A total of 73 HBsAg+/HBsAb+ patients (CHB = 36, HCC = 37) and 96 HBsAg+/HBsAb− patients (CHB = 47, HCC = 49) were enrolled from 13 medical centers in China. The sequence features were elaborated based on the combination of next-generation sequencing (NGS) and multidimensional bioinformatics analysis.

**Results:**

The 16 high-frequency missense mutations, changes of stop codon mutation, clustering, and random forest models based on quasispecies features demonstrated the significant discrepancy power between HBsAg+/HBsAb+ and HBsAg+/HBsAb− in CHB and HCC, respectively. The immunogenicity for cytotoxic T lymphocyte (CTL) epitope Se and antigenicity for the major hydrophilic region (MHR) were both reduced in HBsAg+/HBsAb+ patients (CTL Se: p < 0.0001; MHR: p = 0.0216). Different mutation patterns were observed between HBsAg+/HBsAb+ patients with CHB and with HCC. Especially, mutations in antigenic epitopes, such as I126S in CHB and I126T in HCC, could impact the conformational structure and alter the antigenicity/immunogenicity of HBsAg.

**Conclusion:**

Based on NGS and bioinformatics analysis, this study indicates for the first time that point mutations and quasispecies diversities of HBV s gene could alter the MHR antigenicity and CTL Se immunogenicity and could contribute to the concurrent HBsAg+/HBsAb+ with different features in HCC and CHB. Our findings might renew the understanding of this special serological profile and benefit the clinical management in HBV-related diseases.

## Introduction

Hepatitis B virus (HBV) is a global public health burden that plays an essential role in the development of cirrhosis and liver cancer (1). An estimated 2 billion people worldwide have been infected with HBV, and approximately 250 million are chronic hepatitis B (CHB). Globally, the proportion of hepatocellular carcinoma (HCC) patients induced by HBV infection is 10%–25% (2). The situation is even more severe in the developing world particularly in Asia and Africa since approximately 80% of HCC is related to HBV infection (3). Among different HBV genotypes, genotype C is the most common in South Asia (4), and it is a higher risk for the occurrence of HCC (5, 6).

Hepatitis B surface antibody (HBsAb) is normally considered as an indicator of immune protective status against HBV infection (7). Theoretically, the simultaneous presence of both positive hepatitis B surface antigen (HBsAg) and HBsAb in circulation should not appear in the same patient. However, this understanding has been challenged by several studies indicating the HBsAg+/HBsAb+ in chronic HBV patients from 2.63% to 8.9% (7–11). The mechanism underlying the simultaneous presence of HBsAg and HBsAb remains unclear and even controversial. Moreover, concurrent HBsAg and HBsAb also increased the risk for the development of HCC in CHB (8, 12). Therefore, it is necessary to investigate the clinical as well as biological implications of HBsAg+/HBsAb+ serological profile, which might help in the precise clinical management such as proper therapeutic interventions and disease monitoring in HBV-related diseases (13).

The HBV genome encompasses four overlapping open reading frames (ORFs). S-ORF can be subdivided further into three coding regions (preS1, preS2, and S) encoding three different lengths of HBV surface antigen. HBsAg is the shortest, which is 226 amino acids (aa) long containing several important antigenic epitopes. In particular, the “a” determinant in HBsAg spanning the region of aa124-147 within the major hydrophilic region (MHR; aa99-169) is the major conformational epitope exposed on the external surface of HBV for the antigen–antibody reaction (14). The antigenicity alteration of HBsAg could influence the recognition and neutralization (NT) between HBsAg and HBsAb (15, 16). Several previous studies have attributed the HBsAg+/HBsAb+ serological patterns to aa substitutions that occurred in the “a” determinant of HBsAg resulting in immune evasion (10, 17). The mutation of the HBV genome acquired during the disease process led to HBsAb being incapable of binding mutated HBsAg (18–20). Moreover, viral quasispecies refers to a viral population consisting of extremely large numbers of mutations, termed mutant spectra or mutant clouds (21). HBV strains in infected patients were considered to be a mixture of different quasispecies including mutated HBV and wild-type isolates (22, 23). The existence and expansion of mutated quasispecies might cause the coexistence of HBsAg and anti-HBs antibodies leading to the selection of immune escape (24).

Most studies investigated HBsAg+/HBsAb+-associated mutations on individual sequences derived by the Sanger sequencing or PCR. Due to the technical limitation, rare variants and the vital information within these non-dominant sequences might be ignored. Next-generation sequencing (NGS) is a powerful technique to study quasispecies diversity in viral strains (25). In particular, its high coverage makes its sensitivity much superior to that of traditional methods and offers a powerful method for detecting minor variants as low as 0.25%–5% (26, 27). But till now, only a few studies have utilized NGS towards this special HBV serological status (28–30). Even for those studies, the interpretation of sequence datasets and bioinformatics methods applied to handle such high-dimensional NGS data remains insufficient (31). In addition, the similarity and difference in this special serological pattern for CHB and HCC patients in a large cohort have not been explored.

The big data from NGS have substantial advantage to improve our understanding of the coexistence of HBsAg and HBsAb more comprehensively. Therefore, this study aimed to conduct high-throughput parallel sequencing for a systematic characterization of the HBV s gene between HBsAg+/HBsAb+ and HBsAg+/HBsAb−. To our knowledge, this is the first study to utilize HBV quasispecies features interpreting the underlying mechanism for patients with concurrent presence of positive HBsAg and HBsAb in both CHB and HCC patients.

## Materials and Methods

### Study Population

A total of 364 patients were initially enrolled from December 2015 to June 2020 from the Multi-Center Cooperation Platform of Molecular Diagnostics in China (http://www.multico.com.cn/). The thirteen medical centers of the platform volunteered and were qualified to participate (**Table S1**). The entry criteria of the HCC patients include the diagnosis of HCC based on histology and/or CT/MRI examination (32). Patients with CHB were defined as HBsAg seropositive and/or HBV DNA positive ≥6 months (33). A total of 195 patients were excluded, due to low sequencing depth (<500 reads, 39 cases), non-HBV genotype C (108 cases), and unmatched gender and age (48 cases). Finally, among 169 enrolled patients, 73 subjects infected with HBV genotype C were HBsAg+/HBsAb+ who were assigned to the double-positive (DP) group (CHB = 36, HCC = 37). In order to avoid cohort bias between the DP and single-positive (SP) groups, 96 patients with HBsAg+/HBsAb− whose demographic and clinical characteristics were matched with the DP group were enrolled as the SP group (CHB = 47, HCC = 49). No significant differences between the SP and DP groups in CHB or HCC in terms of variables such as age, gender, total bilirubin (TBIL), total protein (TP), albumin (ALB), alanine transaminase (ALT), aspartate aminotransferase (AST), prothrombin time (PT), and alpha-fetoprotein (AFP) (all p-values >0.05, **Table 1**). The schematic diagram on study design, sequencing design, and data analysis procedure is shown in **Figure S1**.

**Table 1:** Baseline characteristics of CHB and HCC patients in SP and DP groups.

Variables	CHB		p-Value	HCC		p-Value
	SP (n = 47)	DP (n = 36)		SP (n = 49)	DP (n = 37)	
Age (years)	51 (40–73)	54 (40–68)	0.170	54 (40–77)	59 (37–72)	0.300
Gender (%)			0.141			0.919
Male	38 (80.85)	24 (66.67)		42 (95.92)	32 (86.49)	
Female	9 (19.15)	12 (33.33)		7 (4.08)	5 (13.51)	
HBV-DNA (log IU/ml)	5.73 (2.34–8.70)	5.59 (2.04–8.50)	0.977	4.75 (2.05–8.07)	5.79 (2.62–7.73)	0.028
TBIL (μmol/L)	22.2 (5.5–673.5)	20.2 (6.0–461.0)	0.924	17.0 (7.3–431.5)	20.1 (7.2–108.4)	0.156
TP (g/L)	66.4 (20.0–87.9)	71.0 (54.0–86.0)	0.104	68.3 (57.0–87.0)	71.0 (44.3–86.0)	0.918
ALB (g/L)	38.6 (3.9–48.2)	40.1 (19.8–48.5)	0.129	39.7 (31.0–51.5)	40.3 (23.3–73.0)	0.890
ALT (U/L)	56 (12–1,897)	70 (13–830)	0.479	43 (17–1,323)	46 (14–146)	0.744
AST (U/L)	57 (18–2,162)	61 (18–478)	0.715	42 (16–1,526)	49 (21–159)	0.331
PT (s)	13.8 (10.3–37.6)	14.7 (13.1–24.0)	0.727	12.3 (10.7–31.6)	12.2 (10.5–25.3)	0.949
AFP (μg/L)	4.7 (2.0–1,784.0)	7.0 (1.4–2,551.0)	0.311	59.5 (1.3–151,031.0)	185.1 (2.1–882,000.0)	0.117

Categorical variables are presented as number (percentage), and continuous data are presented as median (range). Categorical variables were compared using the chi-square test or Fisher’s exact test. Continuous variables were compared using the independent t-test or Mann–Whitney U test.

SP, single positive (HBsAg+/HBsAb−); DP, double positive (HBsAg+/HBsAb+); TBIL, total bilirubin; TP, total protein; ALB, albumin; ALT, alanine aminotransferase; AST, aspartate aminotransferase; PT, prothrombin time; AFP, alpha-fetoprotein.

### Serum HBV-DNA, HBsAg, and HBsAb Detection

Serum HBV-DNA was quantified using the fluorescence quantitative PCR kit for HBV nucleic acid amplification (Shanghai Kehua Bio-engineering Co., Ltd, China) with a lower limit of detection of 50 IU/ml. HBsAg and HBsAb were detected by the Cobas e602 (Roche, Switzerland). HBsAg >1 COI/ml and HBsAb >10 IU/L were both considered as positive.

### HBV Sequencing and Data Processing

For each serum HBV-DNA of samples, HBV s region (nt273–753) sequencing was performed on the MiSeq sequencer with the MiSeq Reagent Kit, v3 (Illumina, San Diego, CA, USA) using Illumina paired-end sequencing protocols as we established and optimized previously (34). **Figure S2** describes the schematic diagram indicating our studied fragment (s41–s199) in the whole HBV s region (s1–s226). Raw reads were processed by cutadapt 1.15 to cut adaptor sequences and trim low-quality reads (base quality Q20). Filtered read pairs were aligned to the HBV genotype C reference genome sequence (accession numbers: X04615, AY123041, and AB014381) using bwa 0.7.17.

### Quasispecies Characteristics Analysis

The viral quasispecies heterogeneity was mainly evaluated based on genetic complexity and diversity. The complexity was shown by the BioCircos.js tool (35). Quasispecies diversity was evaluated using MEGA X (36) with three parameters: the mean genetic distance (d) of the HBV sequence using Kimura two-parameter model, the number of synonymous substitutions per synonymous site (dS), and the number of non-synonymous substitutions per non-synonymous site (dN) of the HBV sequence using modified Nei–Gojobori method (Jukes–Cantor). To make the calculation feasible, 3,000 reads were randomly chosen from each sample by replicating three times. Subsequently, the complexity of each nucleotide and amino acid position in s region was calculated based on five sequence features including ACGT (nt), Mutation (nt), Entropy (nt), Mutation (aa_s), and Entropy (aa_s) established by our previous research (34). Additionally, mutations were identified and analyzed using R scripts. High mutation frequency was defined as a mutation rate ≥5% of the total reads in each position. Statistical significance was evaluated using the unpaired Wilcoxon test and Fisher’s exact test to identify differential mutations between the SP and DP groups in the CHB and HCC patients, respectively.

### Epitope Analysis

To predict the immunogenicity of HBV cytotoxic T lymphocyte (CTL) epitopes (Sb–Se) (24), immunogenicity score (IS) was established using Class I Immunogenicity Python script (37) based on the immunogenicity model defined by Calis et al. (38) on the Immune Epitope Database (IEDB). Similarly, the antigenicity score (AS) of the HBV MHR was predicted based on the Kolaskar and Tongaonkar antigenicity scale on IEDB (39). For each sample i, depth_i_ refers to the sequencing depth (total number of reads) of sample i. IS_REF_/AS_REF_ refers to the IS/AS value of the reference sequence. Thus, the decreasing percentage of IS (dp.IS) and AS (dp.AS) was defined as follows:


(1) $$\text{dp}.{\text{IS}}_{\text{i}}=\frac{1}{{\text{depth}}_{\text{i}}}\underset{1\leq \text{j}\leq {\text{depth}}_{\text{i}},{\text{ IS}}_{\text{j}}<{\text{IS}}_{\text{REF}}}{\sum }1$$

(2) $$\text{dp}.{\text{AS}}_{\text{i}}=\frac{1}{{\text{depth}}_{\text{i}}}\underset{1\leq \text{j}\leq {\text{depth}}_{\text{i}},{\text{ AS}}_{\text{j}}<{\text{AS}}_{\text{REF}}}{\sum }1$$

### Analysis of Physical Properties and Construction of 3D Model

Hydrophilicity and transmembrane tendency scores for each residue were determined by using the ExPASy ProtScale website (4, 40). To compare the 3D structure of MHR amino acid residues associated with different mutation patterns of site 126, three 3D models—MHR-WT, MHR-I126S, and MHR-I126T—were generated *in silico* using Robetta server (41) and visualized by Pymol2.4.

### Neutralization Rate Experiment

NT test was performed in reference to previous research (7). Sera from five healthy subjects who had received recombinant HBV vaccine were collected and pooled. We measured the HBsAb level on Cobas e602 system (Roche, Switzerland); then available sera of the DP patients (HCC = 29, CHB = 30) were incubated with diluted vaccine serum at 37°C for 1 h. After NT, the HBsAg level was detected on the Roche system. The NT rate formula was as follows:


(3) $$NT\;rate=\frac{HBsAg\;concentration_{before\;neutralization}-HBsAg\;concentration_{after\;neutralization}}{HBsAg_{before\;neutralization}}\times 100\%$$

## Results

### Complexity/Diversity of HBV s Region Between Single-Positive and Double-Positive Patients

**Figure 1:**
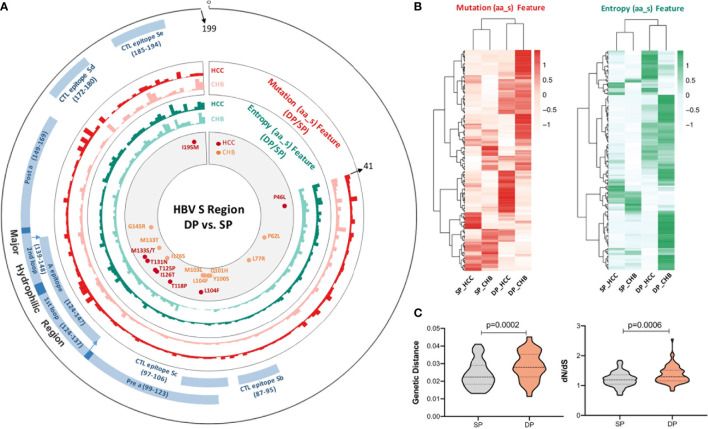
The complexity and diversity of HBV s region between double-positive (DP) and single-positive (SP) patients in chronic hepatitis B (CHB) or hepatocellular carcinoma (HCC) patients. **(A)** The outermost circle represents the location of each functional region of the HBsAg protein (s41–199). The important functional HBV s region distribution was marked as blue and as follows: major hydrophilic region (MHR, s99–169) overlap of Pre a (s99–123), 1st loop (s124–137), A epitope (s124–147), 2nd loop (s139–148), and Post a (s149–169); CTL epitope Sb (s87–95), CTL epitope Sc (s97–106), CTL epitope Sd (s172–180), and CTL epitope Se (s185–aa194). The colored histograms in the second and third circles indicate the complexity of mutation feature (dark red, HCC; light red, CHB) and entropy feature (dark green, HCC; light green, CHB) for each amino acid, respectively. The histogram value represents the feature ratio of DP to SP. The innermost circle shows the differential high-frequency mutations between DP and SP groups for both CHB (orange) and HCC (red) patients, respectively. **(B)** Different HBV s region features among SP_HCC, SP_CHB, DP_HCC, and DP_CHB groups by hierarchical clustering. The value in the clustering map represents the mutation (red, left) and entropy (green, right) features of each group. A correlation was used for the sample measurement, Euclidean distance for the feature measurement, and ward.D2 algorithms for the clustering method. **(C)** Different HBV s region quasispecies characteristics between SP (gray) and DP (orange) patients using MEGA X. The y-axis of the left violin plot represents the number of genetic distance (d), and the right represents the ratio of non-synonymous substitutions per non-synonymous site (dN) to the number of synonymous substitutions per synonymous site (dS). Statistical significance was evaluated using the unpaired Wilcoxon test.

Differential sequence characteristics (complexity and diversity) between the SP and DP patients in both CHB and HCC were observed, respectively. **Figure 1A** shows that mutation and entropy features of the DP patients were higher than those of SP patients in several important regions of the studied fragment (s41–199), including the MHR, CTL epitopes (Sb–Se), and the N-terminal region. In addition, the distribution of high feature ratio (DP/SP) in s41–199 varied between CHB and HCC groups (**Figure 1A**). Interestingly, cluster analysis showed that sequence features within DP or SP patients (DP_CHB vs. DP_HCC or SP_CHB vs. SP_HCC) were more similar, regardless of whether the disease status was CHB or HCC (**Figure 1B**). Additionally, the quasispecies diversity distribution has significant differences between the DP and SP groups (**Figure 1C**). The DP group had higher mean genetic distance (d) and higher ratio of dS to dN (dS/dN) in the HBV s region (both p-values <0.001). The above results indicated the significant discrepancies in quasispecies characteristics between DP and SP in both the CHB and HCC patients.

### Differential High Frequencies of Amino Acid Mutations Between Double Positive and Single Positive

**Figure 2:**
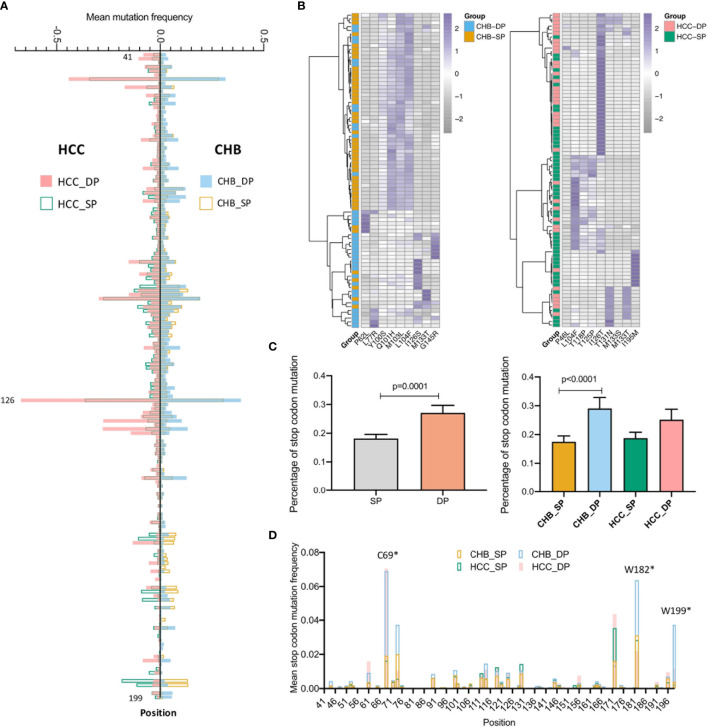
Comparison of differential mutations between double-positive (DP) and single-positive (SP) groups in chronic hepatitis B (CHB) and hepatocellular carcinoma (HCC) patients. **(A)** The barplot demonstrates differential average mutation frequency of each position in the HBV s41–199 region between SP and DP groups for CHB and HCC patients. **(B)** The majority of DP and SP patients in both CHB and HCC can be well-classified by the hierarchical clustering based on high-frequency mutation sites, which were defined as a mutation rate ≥5% of the total reads in each position and differential mutated between SP and DP groups in CHB and HCC patients using both the unpaired Wilcoxon test and Fisher’s exact test. **(C)** The top histogram demonstrates different percentages of stop codon mutations in HBV s41–199 region between all SP and DP patients, and the bottom represents different percentage of stop codon mutations in subgroups among CHB_SP (yellow), CHB_DP (light blue), HCC_SP (green), and HCC_DP (pink) patients. Statistical significance was evaluated using the unpaired Wilcoxon test. **(D)** The barplot demonstrates differential average stop codon mutation frequency of each position in the HBV s41–199 region between SP and DP groups for CHB and HCC patients. *represents stop codon mutation.

The distribution of average mutation frequency of each site in the studied fragment between the SP and DP groups varied in both CHB and HCC (**Figure 2A**). The mutation frequency of all positions was compared between the SP and DP groups with a cutoff of no less than 5% for each site. Sixteen differential high frequencies of missense mutations were identified (p < 0.05), including P46L, P62L, L77R, Y100S, Q101H, M103L, L104F, T118P, T125P, I126S/T, T131N, M133T/S, G145R, and I195M. Among these 16 mutations, 12 mutations are in the MHR of HBsAg, which might contribute to viral immune escape. Among them, L104F and M133T were both identified in the CHB and HCC patients. Furthermore, P62L, L77R, Y100S, Q101H, M103L, I126S, and G145R mutations were found only in CHB, whereas P46L, T118P, I126T, T125P, T131N, M133S, and I195M only occurred in HCC. Interestingly, hierarchical clustering revealed that the majority of the DP and SP patients in CHB or HCC were clustered together based on mutation frequencies of the corresponding identified mutations (**Figure 2B**). Moreover, stop codon mutations were observed more frequently in the DP groups than in the SP groups (**Figure 2C**, p = 0.0001). Especially, in the CHB patients, percentages of stop codon mutations were significantly higher in the DP group (p < 0.0001). Similarly, a higher stop codon mutation tendency in the HCC_DP group was also observed. Likewise, the site of average stop codon mutation frequency (mutation frequency >0) in the studied fragment was higher in the DP groups than the SP groups, which was confirmed in most sites in both the CHB and HCC patients (**Figure 2D**).

### Efficacy of Feature Patterns in Distinguishing Double-Positive From Single-Positive Patients

**Figure 3:**
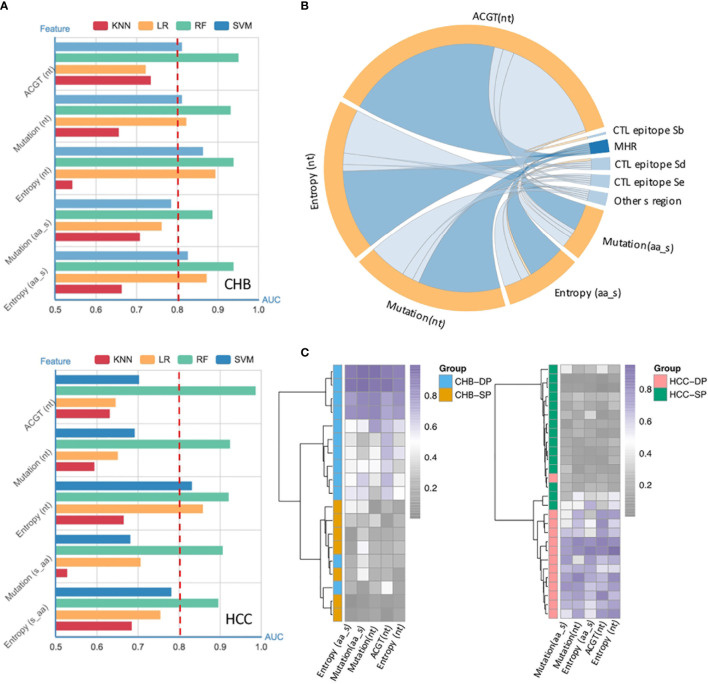
Performance of quasispecies feature patterns to distinguish double-positive (DP) from single-positive (SP) patients. **(A)** High performance of the HBV random forest (RF) model for DP prediction in chronic hepatitis B (CHB) (up) and hepatocellular carcinoma (HCC) (down) patients. Performance of four models used to identify and classify SP and DP patients in CHB (up) and HCC (down), based on five types of significant features extracted from the HBV fragment (nt273–753) by fivefold cross-validation. Significant features were identified by the differential feature analysis between SP and DP groups, and both p-values of <0.05 and area under the receiver operating characteristic curve (AUC) >0.8 were considered significant. The x-axis represents the value of the AUC, and the y-axis indicates different features [ATCG; mutation and entropy of HBV nucleotide sequence nt273–753; mutation and entropy feature of HBsAg amino acid sequence s41–199]. Different colors represent different machine-learning models (RF, random forest; SVM, Support Vector Machine; kNN, k-nearest neighbor; LR, logistic regression). **(B)** High enrichment of significant features in the HBV major hydrophilic region (MHR). Significant features were derived from differential feature analysis between SP and DP groups as mentioned above. The chord diagram indicates connections between significant features and different HBV functional regions, which were represented by fragments (nodes) on the outer part of the circular layout (features, orange; HBV regions, blue). The arc length of each fragment indicates the count of significant features. The internal connection band indicates the flow direction of the data relationship. The darker blue color indicates that more significant features were enriched in MHR. **(C)** Most poorly distinguished DP and SP patients (CHB, n = 19; HCC, n = 28) derived from a high frequency of mutation site-based clustering can be well-classified using HBV RF model based on five types of sequence features.

Our previous study has demonstrated that vital HBV quasispecies features showed a promising clinical potential for improving HCC prediction (34). To determine whether feature patterns extracted from studied fragments could also have a good discrimination power between the SP and DP patients, binary classification was performed by the cross-validation of four machine learning models. Interestingly, the random forest (RF) model performed best in differentiating the DP group based on all five types of features as described previously (see *Materials and Methods*), with an, area under the receiver operating characteristic curve (AUC) of approximately 0.9 irrespective of disease types (**Figure 3A**). Moreover, to better visualize the connection between feature patterns and HBV functional regions, a chord diagram was created. **Figure 3B** demonstrates that the HBV MHR showed the strongest relationship with these five features than other CTL epitopes and other s regions of HBV. To further assess whether feature patterns could enhance classification accuracy, the RF model was trained based on patients who can be well-classified on the basis of the hierarchical clustering (**Figure 2B**). And then the trained RF model was utilized to discriminate DP between SP patients. Intriguingly, it was found that 47 patients (CHB, n = 19; HCC, n = 28) who were not assigned to their correct clusters *via* hierarchical clustering based on the differential high-frequency mutations can be basically well-classified. Furthermore, clear differences in terms of quasispecies features between the DP and SP patients were seen in both CHB and HCC (**Figure 3C**). All five types of significant feature patterns exhibited a great predictive power between SP and DP, in both the CHB patients (AUCs: 0.77–1.0) and HCC patients (AUCs: 0.89–0.97) (**Figure S3**). This suggested that sequence data with feature patterns can provide more information than mutation alone to improve the performance in distinguishing the DP patients.

### Reduced Antigenicity/Immunogenicity of the s Region in Double-Positive Patients

**Figure 4:**
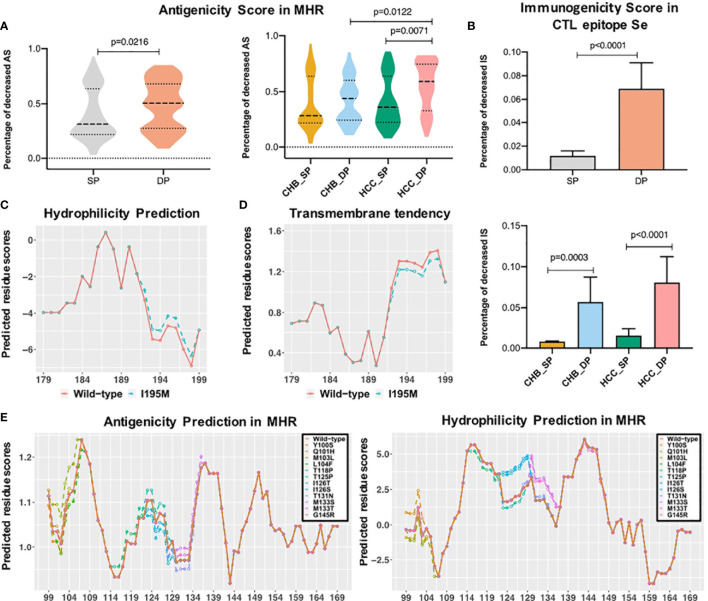
Epitope analysis between double-positive (DP) and single-positive (SP) group in chronic hepatitis B (CHB) and hepatocellular carcinoma (HCC) patients. **(A)** The violin plot demonstrates that decreased antigenicity score (AS) percentage in the major hydrophilic region (MHR) of DP patients (orange) was higher than that of SP patients (gray), and the bottom plot represents the difference of decreased AS percentage in four subgroups. **(B)** The left histogram plot demonstrates that decreased immunogenicity score (IS) percentage in the CTL epitope Se region of DP patients (orange) was higher than that of SP patients (gray), and the right plot represents the difference of decreased IS percentage in subgroups among CHB_SP (yellow), CHB_DP (light blue), HCC_SP (green), and HCC_DP (pink) patients. Statistical significance was evaluated using the unpaired Wilcoxon test. **(C)** I195M mutation increased the hydrophobicity of the third trans-membrane HBsAg domain for HCC patients. The x-axis represents the position of the third transmembrane region, and the Parker hydrophilicity score (y-axis) was predicted for each residue by using the IEDB website. The orange line refers to wild type, and the green dotted line represents I195M mutation. **(D)** I195M mutation reduced the transmembrane tendency of the third trans-membrane HBsAg domain for HCC patients. The transmembrane tendency score (y-axis) was determined for each residue by using the ExPASy ProtScale website. **(E)** Differential mutations identified between SP and DP groups influenced the antigenicity and hydrophobicity of the MHR. The prediction score (y-axis) was determined for each residue by the IEDB website using the Kolaskar and Tongaonkar antigenicity scale and Parker hydrophilicity method. The orange line refers to wild type, and other colored lines represent different high frequencies of mutations.

The MHR of the HBV s region contains high-conformation clusters of B cell epitopes, which is the main target of HBsAg neutralizing antibodies (42, 43). To observe the impact of mutations on the antigenicity of HBsAg in each patient, the dp.AS in HBV MHR was calculated. Considerably increased dp.AS in the DP patients was found in the MHR of HBV (**Figure 4A**, p = 0.0216). For the CHB and HCC subgroups, a reduced proportion of AS was statistically greater in the HCC_DP groups (HCC_SP vs. HCC_DP: p = 0.0071, CHB_DP vs. HCC_DP: p = 0.0122). Likewise, there is a tendency for the patients in the CHB_DP group to have a greater dp.AS than those in the CHB_SP group (**Figure 4A**). Previous research pointed out that mutations of both B-cell and - cell epitopes can explain the coexistence of HBsAg and HBsAb. In genotype C patients, mutations in the CTL epitope of the HBV s region resulted in higher aa mutations than the HBsAg group alone and impaired the recognition sites of immune cells leading to the prevention of the CTL activation (24). Therefore, the response change of cellular immunity against the HBV antigen also cannot be underestimated. Notably, our result corroborated that significantly increased dp.IS in the DP patients was found in CTL epitope Se (**Figure 4B**, p < 0.0001), and these results are also confirmed within the subgroups (CHB: p = 0.0003, HCC: p < 0.0001).

### Impact of Mutations on the Hydrophilicity Profile in Double-Positive Patients

Most identified mutations associated with the coexistence of HBsAg and HBsAb are localized in the cytoplasmic domain of the HBsAg. In particular, the residue at s195 is embedded in the third trans-membrane HBsAg domain. The change of sequence properties in these two domains may influence the proper conformation of different forms of HBV surface antigen. Thus, to make a more detailed analysis, calculations of hydrophilicity and transmembrane tendency were performed in this study. **Figures 4C, D** indicate that I195M leads to higher hydrophilicity and lower transmembrane tendency. Moreover, the antigenicity and hydrophilicity scores for different mutations that existed in the cytoplasmic domain of HBsAg was further calculated. Particularly, I126S and I126T mutations were associated with obvious lower antigenicity and higher hydrophilicity scores, compared with the wild-type amino acid (**Figure 4E**). The change of sequence properties in these two domains may influence the proper conformation of the different forms of HBV surface antigen.

### Different Mutation Patterns of the Amino Acid s126 in Double-Positive Patients

**Figure 5:**
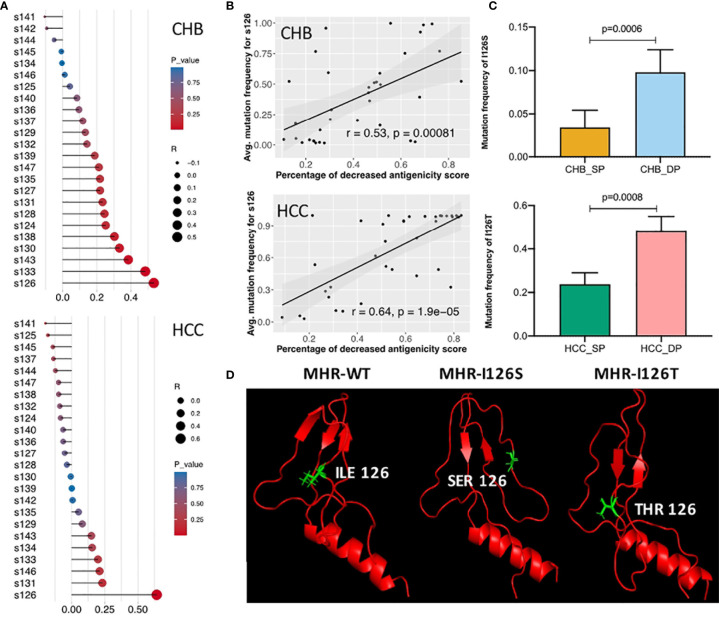
Identification of different mutation patterns of s126 in double-positive (DP) patients. **(A)** Correlation analysis among the decreased percentage of antigenicity score (AS) (dp.AS) and mutation frequency of each position in the major hydrophilic region (MHR) A epitope (s124–147). The lollipop plot was used to visualize and indicate Spearman’s correlation coefficient. The length of the line and the size of the circle represent the absolute value of the correlation R, and the color of the circle represents the p-value. **(B)** High positive correlation was shown between dp.AS in the MHR A epitope and average mutation frequency of position s126 in patients with chronic hepatitis B (CHB) (up) and hepatocellular carcinoma (HCC) (down), respectively. **(C)** Different mutation patterns of position s126 in patients with CHB (up) and HCC (down). The top histogram plot represents the mutation frequency of I126S in CHB_SP (yellow) and CHB_DP (light blue), and the bottom histogram represents the mutation frequency of I126T in HCC_SP (green) and HCC_DP (pink) patients. Statistical significance was evaluated using the unpaired Wilcoxon test. **(D)** Three 3D prediction structures of MHR amino acid residues associated with different mutation patterns of position s126 (left, wild type; middle, I126S; right, I126T). The chains of s126 were labeled as sticks and marked as green color.

Correlation analysis was further performed between dp.AS and mutation frequency of each site in the MHR A epitope (**Figure 5A**). A high positive correlation was shown between dp.AS and average mutation frequency of s126 in both CHB (r = 0.53, p = 0.00081) and HCC (r = 0.64, p < 0.0001) (**Figure 5B**). More interestingly, a different mutation pattern of s126 was observed between patients with CHB and with HCC (**Figure 5C**). The CHB patients had a higher average mutation frequency for I126S mutations in the DP group (p = 0.0006), while the HCC patients had I126T mutation with an average mutation frequency over 40% in the DP group, which was much greater than that in the SP group (p = 0.0008). A further study was conducted to provide protein 3D structure prediction of MHR amino acid residues associated with different mutation patterns of s126. In three 3D models spanning from s99 to s169 (MHR-WT, MHR-I126S, and MHR-I126T), changes of MHR-I126S and MHR-I126T models could affect the proper tertiary structure of HBsAg, indicating their possible roles in an alteration of immunogenicity of HBsAg (**Figure 5D**).

### The Association Between Entropy Features and Clinical Routine Tests in Double-Positive Patients

**Figure 6:**
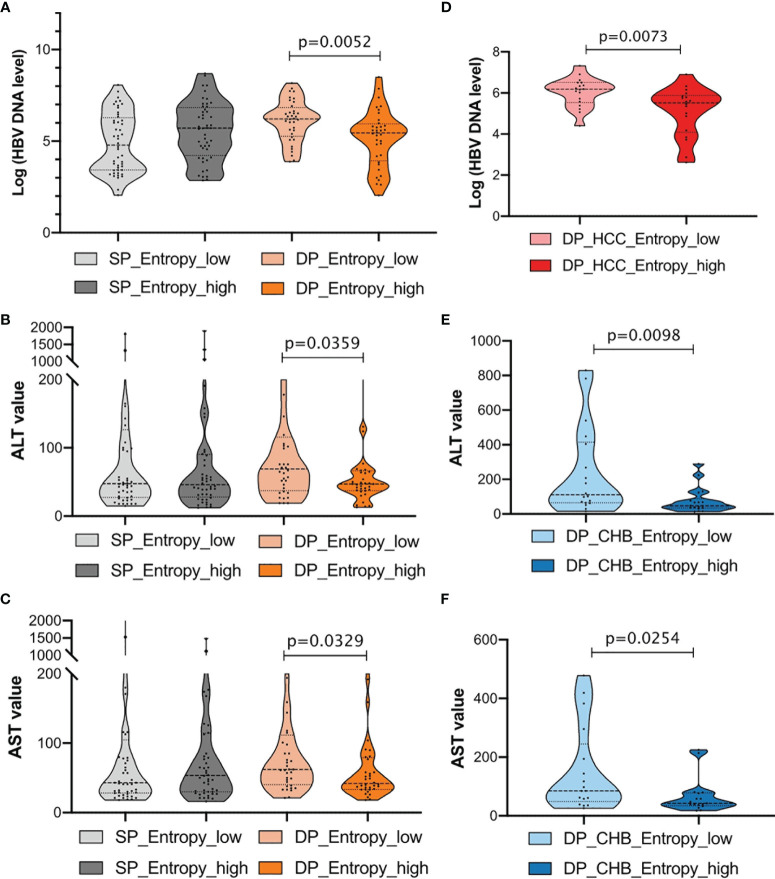
Clinical characterization in double-positive (DP) patients. **(A–C)** Comparison of HBV DNA log level, alanine transaminase (ALT), and aspartate aminotransferase (AST) values between single-positive (SP) and DP groups. Patients were further classified into two subgroups, respectively, according to the sum of entropy feature (high, top 50%; low, bottom 50%). The entropy feature based on nucleotide level was marked below, and statistical significance was evaluated using the unpaired Wilcoxon test. **(D)** Violin plots demonstrate the relationships between the entropy feature and HBV DNA log levels in HCC_DP groups. Patients were classified into two subgroups according to the sum of entropy feature (high, top 50%, dark red; low, bottom 50%, light red). **(E, F)** Violin plots demonstrate the relationships between the entropy feature and ALT or AST values in CHB_DP groups, separately. Patients were classified into two subgroups according to the sum of entropy feature (high, top 50%, dark blue; low, bottom 50%, light blue).

To further explore the association between clinical indicators and sequence features, patients were then classified into two subgroups, respectively, stratified by 50% of the sum of entropy features. Notably, we found that patients with high entropy features have significantly lower HBV DNA log, ALT, and AST levels in the DP samples, whereas there is no obvious difference in the SP samples (**Figures 6A–C**). For the CHB and HCC subgroup analysis in the DP samples, it was found that higher entropy features were associated with lower HBV DNA levels in the HCC_DP patients (**Figure 6D**), and lower entropy features were related to higher ALT and AST values in the CHB_DP patients (**Figures 6E, F**). This result further demonstrated that feature patterns acquired by NGS are closely associated with clinical demographic changes in the DP samples.

## Discussion

This study performed a comprehensive exploration of characteristics and diversities of HBV s region in the CHB/HCC patients with the coexistence of HBsAg and HBsAb. The systematic analysis includes high frequency of mutations, quasispecies patterns, antigenicity/immunogenicity, physical properties, and clinical characterization. This research for the first time investigated the similarity and difference of HBV s gene features between DP and SP in both the CHB and HCC patients. More importantly, it further revealed the selection of HBsAg immune escape mechanism from the HBsAb based on a large-representative multicenter NGS cohort and powerful bioinformatics analysis.

The study showed that the DP group accumulated more variants than the SP group in HBsAg. It was observed that higher mutations are harbored in the s region, which was in line with the previous researches (44, 45). This study discovered two mutations, L104F and M133T, in both the CHB_DP and HCC_DP patients. The DP profile in CHB is associated with accumulative HBsAg mutations P62L, L77R, Y100S, Q101H, M103L, I126S, and G145R, whereas P46L, T118P, I126T, T125P, T131N, M133S, and I195M are harbored in HCC. Moreover, the presence of mutations in and around antigenic epitopes, such as I126S, I126T, and I195M, may impact the conformational structure of this region, hence altering the antigenicity/immunogenicity of HBsAg and leading to immune escape in the end (**Figures 4C–E**) (46). This study identified that the DP profile showed different mutation patterns of s126 in patients between CHB and HCC. Identified mutation I126S in CHB and I126T in HCC were both correlated with the alteration of HBsAg antigenicity (**Figure 5**). Moreover, the NT experiment was performed to validate the impact of the mutation frequency of I126S in CHB_DP or I126T in HCC_DP on the NT rate. The result showed that the NT rate has a reduced trend in the DP patients with I126S or I126T (**Figure S4**). In addition, due to the overlap between HBsAg and rt gene, I195M, corresponding to rt changes of M204V/I, is considered as a lamivudine-resistant mutation, which may also raise the possibility of drug-selected HBsAg and the potential to escape NT by HBsAb (47).

Besides missense mutations, the percentage of stop codon mutations identified by NGS was also found much higher in the DP patients than in the SP patients. The proportion of W199* in the DP groups was found to be significantly greater than that in the SP groups. Moreover, the average mutation frequency of C69* was much higher in both the CHB_DP and HCC_DP patients. Higher stop codon mutations could not only abrogate full-length wild-type HBsAg quantification to affect the proper diagnosis of disease severity (48) but also reduce the efficient recognition and NT by HBsAb. Both two mutations have been described to contribute to the progression of HCC (49, 50). Besides, W182* rate in the CHB_DP group was significantly higher than the rates in the CHB_SP group, whereas no obvious difference was observed in patients with HCC. It was reported that premature stop at codon 182 may provide an important contribution to the progression of HCC (51), which might explain the differences of W182* between the CHB_DP and HCC_DP groups.

Our results suggest that viral quasispecies features in the s gene play an important role in the development of DP profile. The DP profile was partially associated with the emergence of HBV escape mutations (**Figure 2**). Meanwhile, there were yet some patients who failed to be clustered together by several high frequencies of amino acid substitutions in HBsAg. Interestingly, when NGS was applied to characterize HBV quasispecies, it was found that five quasispecies features patterns can well classify those patients who were misclassified based on differential mutations (**Figure 3**). This result suggests that quasispecies features provide a better indication of HBsAg+/HBsAb+ than mutations. Entirely, the distribution of feature patterns was complex in the DP patients but relatively simple for SP patients, which may be attributed to the diversity of HBV quasispecies (**Figure 1**).

The advantage of quasispecies features in interpreting the immune escape in the DP patients was further studied. Data from NGS revealed that the genetic distance, as well as the ratio of dN/dS, varied between the DP and SP groups (**Figure 1**). The mean viral genetic distance was greater in the DP group (p = 0.0002) combined with a higher dN/dS ratio (p = 0.0006), indicating stronger positive selection acting on HBsAg to enhance viral fitness (52). These findings provided clues for differences in selection pressures between the SP and DP patients. Besides, decreased immunogenicity of CTL epitopes in DP might also contribute to immune escape (53). As MHR A epitope and CTL epitopes situated on the surface of HBV have been described to be potential NT domains, we can speculate that increased genetic diversity may lead to altered T-cell and B-cell epitopes, which can change their immunogenicity leading to escape from host immune response (54). Thus, we further investigated the change of antigenicity/immunogenicity in these regions to study the coexistence mechanism of HBsAg+/HBsAb+. Notably, our findings demonstrated that the percentages of antigenicity for MHR and immunogenicity for CTL epitope Se (**Figures 4A, B**) were both reduced in the DP patients. To study whether the accumulation of hotspot mutations in the DP patients can contribute to immune evasion, we then compared the change of dp.AS between high accumulation of hotspot mutations (defined as the total mutation rate of differential high-frequency mutation sites) and low accumulation of hotspot mutations in the DP samples. We found that a considerably reduced dp.AS in the DP patients was shown in the MHR of HBV in the low accumulation of hotspot mutations group, compared with the high accumulation of hotspot mutation group (**Figure S5**, p = 0.0128). For the CHB_DP and HCC_DP subgroups, there is a consistent tendency that patients in the CHB_DP and HCC_DP groups were found to have a greater dp.AS in high accumulation of hotspot mutation groups (**Figure S5**).

More interestingly, our study demonstrated that entropy features are also associated with the clinical indicators of HBV DNA duplication and liver inflammation for the DP samples. Previous studies (55–57) have observed a negative relationship between viral diversity (Shannon entropy) and HBV DNA level in s region. Our study further indicated this inverse relationship firstly in the DP samples. One possible explanation could be that viruses adapted to host immune pressure at the cost of viral replication, which imperils the efficient HBV DNA replication (55). We speculated that strong host immune stress suppressed viral replication. Meanwhile, it also increased the selection of mutations in HBsAg, which as a consequence increases the viral diversity. This might explain the reason why with the accompanying high features of s region in the DP patients, the viremia status was lower. Moreover, we also analyzed the HBsAb level with mutation rates, HBV-DNA, ALT/AST, and entropy features. We performed the correlation analysis between the mutation rate of differential high-frequency mutation sites and HBsAb levels in CHB_DP and HCC_DP, respectively. Among those high-frequency mutation sites, there was a statistically significant correlation between the mutation frequency of site 125 and HBsAb levels in the HCC_DP samples (R = −0.38, p = 0.04) (**Figure S6A**). We further analyzed the correlation between the total mutation feature value of s gene and the HBsAb levels. In the HCC_DP samples, there was a tendency that increased s gene mutation feature values were associated with the decreased HBsAb levels (**Figure S6B**). While the current data can only show the tendency, more experiments are needed to validate whether a higher s gene mutation contributes to the decline of HBsAb in the future. Subsequently, we analyzed the difference between the HBsAb level and HBV-DNA, ALT/AST, or entropy features in the DP patients. Although we did not observe the obvious impact of the change of HBsAb level on entropy features and clinical routine tests (**Figure S7**), lower entropy features were associated with higher HBV DNA titer and higher ALT and AST values (**Figure 6**). We further research the HBsAb loss in HBsAg+/HBsAb+ patients. During the follow-up of our study (n = 73), there were only two DP samples with HBsAb loss: case 3 (CHB_DP) and case 6 (HCC_DP). The HBsAb level of case 3 decreased from 10.74 to <0.2 IU/L after 101 days. Likewise, in 2016, the HBsAb level of case 6 was 89.8 IU/L, which decreased to 4.56 IU/L in 2018. Both of the cases were also accompanied by the decreasing HBV DNA (case 3: dropped from 382,000 to <50 IU/ml; case 6: dropped from 15,700 to <50 IU/ml). An analysis of the hot mutations and entropy feature was performed. Several mutants in the MHR were identified, and a low total entropy feature of the MHR was also observed in these two cases.

We also acknowledge certain limitations in this study. Firstly, insertions and deletions were not included due to technical limitations, which may also result in the change of the conformational structure and antigenicity of HBsAg. Secondly, since the HBV genome is compact and contains overlapping genes, mutations in HBsAg can also influence the functions of other encoding regions. The potential of concomitant mutations in these regions might be missed in this study. Therefore, how insertions/deletions and other HBV regions have an impact on the immunogenicity of HBsAg should be evaluated in our further research.

In conclusion, utilizing HBV s region sequence analyses, mathematical models, and immune characterization, this study demonstrated a comprehensive characteristic of viral quasispecies between the SP and DP patients by NGS from a large-representative multicenter cohort. Integration of sequence feature patterns is capable of revealing the controversial underlying mechanism of a mixed viral population in the DP patients, and it contributes to better interpretation of the coexistence of HBsAg and HBsAb and benefits to precise clinical management.

## Data Availability Statement

The datasets presented in this study can be found in online repositories. The names of the repository/repositories and accession number(s) can be found below: https://www.ncbi.nlm.nih.gov/sra/?term=PRJNA765447.

## Ethics Statement

The studies involving human participants were reviewed and approved by the Institutional Ethics Committee of the leading medical center (Shanghai Eastern Hepatobiliary Surgery Hospital, EHBHKY2015-01-004). The patients/participants provided their written informed consent to participate in this study.

## Author Contributions

YW, XX, and SC performed the investigation, methodology, and formal analysis and drafted the manuscript. CH, JZ, and ZG performed data curation and technique assistance. ED, YL, XH, and CW provided the clinical samples. CG obtained the funding, designed and coordinated the overall study, and revised the manuscript with MF. All authors contributed to the article and approved the submitted version.

## Funding

This work was supported by the Innovation Group Project of Shanghai Municipal Health Commission [2019CXJQ03], the National Natural Science Foundation of China [81572072], the Science and Technology Commission of Shanghai Municipality [17JC1404500 and 16441907200], and the Shanghai “Rising Stars of Medical Talent” Youth Development Program [2019-72].

## Conflict of Interest

The authors declare that the research was conducted in the absence of any commercial or financial relationships that could be construed as a potential conflict of interest.

## Publisher’s Note

All claims expressed in this article are solely those of the authors and do not necessarily represent those of their affiliated organizations, or those of the publisher, the editors and the reviewers. Any product that may be evaluated in this article, or claim that may be made by its manufacturer, is not guaranteed or endorsed by the publisher.
