# Supplementary material for: The Impact of HBV Quasispecies Features on Immune Status in HBsAg+/HBsAb+ Patients With HBV Genotype C Using Next-Generation Sequencing
Source: Front Immunol. 2021 Nov 25;12:775461. doi: 10.3389/fimmu.2021.775461 (PMC8656693; doi:10.3389/fimmu.2021.775461)
Supplement: Supplementary file 1 [file DataSheet_1.pdf]

The Impact of HBV Quasispecies Features on Immune Status in HBsAg+/HBsAb+ Patients With HBV Genotype C Using Next-Generation Sequencing

Ying Wang[1#], Xiao Xiao[1,2#], Shipeng Chen[1#‡], Chenjun Huang[1], Jun Zhou[1], Erhei Dai[3], Ya Li[4], Lijuan Liu[5], Xianzhang Huang[6], Zhiyuan Gao[1,2], Chuanyong Wu[2], Meng Fang[1*], Chunfang Gao[1,2*]

[1]Department of Laboratory Medicine, Shanghai Eastern Hepatobiliary Surgery Hospital, Shanghai, 200438, China

[2]Clinical Laboratory Medicine Center, Yueyang Hospital of Integrated Traditional Chinese and Western Medicine, Shanghai University of Traditional Chinese Medicine, Shanghai, 200437, China

[3]Department of Laboratory Medicine, the Fifth Hospital of Shijiazhuang, Hebei Medical University, Hebei, 050021, China

[4]Department of Laboratory Medicine, the First Affiliated Hospital of Kunming Medical University, Yunnan, 650032, China

[5]Department of Laboratory Medicine, Mengchao Hepatobiliary Hospital of Fujian Medical University, Fuzhou, 350025, China

[6]Department of Laboratory Medicine, the Second Affiliated Hospital of Guangzhou University of Chinese Medicine, Guangdong, 501020, China

[‡]Present Address: Department of Medical Microbiology and Infection Prevention, Tumor Virology and Cancer Immunotherapy, University Medical Center Groningen, University of Groningen, Groningen, The Netherlands

#These three authors contributed equally to this work.

***Corresponding:**
Chunfang Gao
gaocf1115@163.com
Meng Fang
fmmeng83@163.com

Table S1. Sample statistics from 13 medical centers in China

ID	Medical Center	Num. of SP	Num. of DP
1	Shanghai Eastern Hepatobilliary Surgery Hospital	47	26
2	Fifth Hospital of Shijiazhuang	0	32
3	First Affiliated Hospital of Kunming Medical University	0	7
4	Mengchao Hepatobilliary Hospital of Fujian Medical University	0	3
5	Beijing You'an Hospital	1	0
6	The Second Affiliated Hospital of Guangzhou University of Traditional Chinese Medicine	2	1
7	Yueyang Hospital of Integrated Traditional Chinese and Western Medicine	0	4
8	Henan Provincial Hospital of Traditional Chinese Medicine	5	0
9	Taizhou First People's Hospital	3	0
10	Jiangsu Provincial People's Hospital	21	0
11	Zhongshan Hospital Affiliated to Xiamen University	5	0
12	The General Hospital of Shenyang Military District	2	0
13	The 211 Hospital of PLA	10	0
	**Total**	**96**	**73**

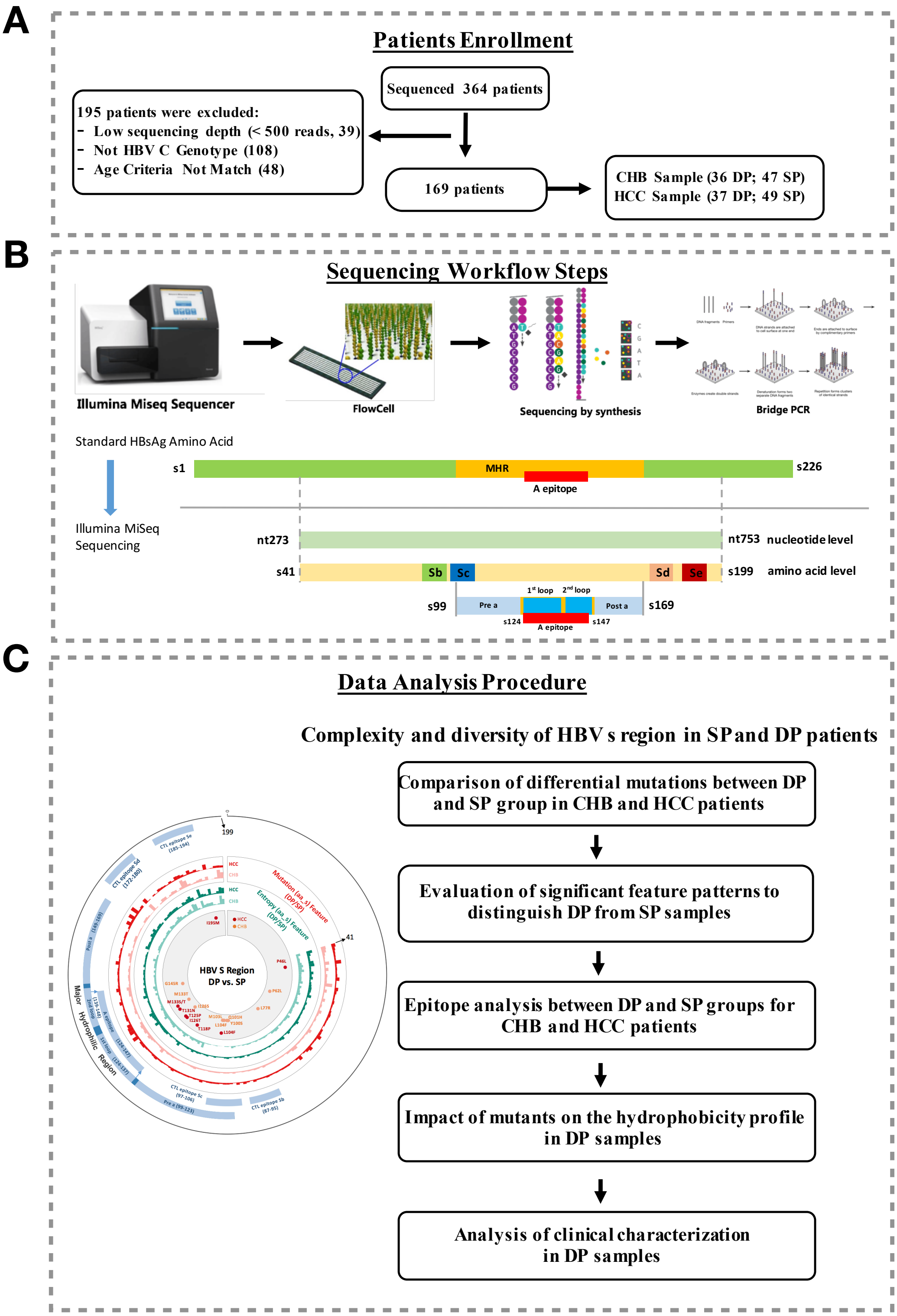

Figure S1. A schematic diagram on research design, sequencing workflow steps and diagnostic prediction model development

A. Details of the study enrollment design. 364 patients were included from 13 multicenter research; 39 patients were excluded due to low sequencing depth (<500 reads). In the remaining patients belong to HBV genotype C, among who 73 DP patients (36 CHB; 37 HCC) were finally enrolled

in this study. In addition, 96 SP patients (47 CHB; 49 HCC) whose age match to DP patients were also selected as proper control groups.

B. NGS sequencing workflow steps of HBV s region. The NGS workflow included HBV genome extraction, amplification of HBV s region by PCR, the purification and quantification, library construction, quality control by quantitative PCR (qPCR), and deep sequencing. For the deep sequencing step, several samples can be loaded onto the flow cell for simultaneous analysis, and then the bridge PCR amplification process was applied.

C. Data analysis procedure of complexity and diversity of HBV s region in SP and DP patients. Differential mutations, feature patters, epitopes, hydrophobicity profile and clinical characterization analyses were performed between DP and SP groups both in the CHB and HCC patients respectively.

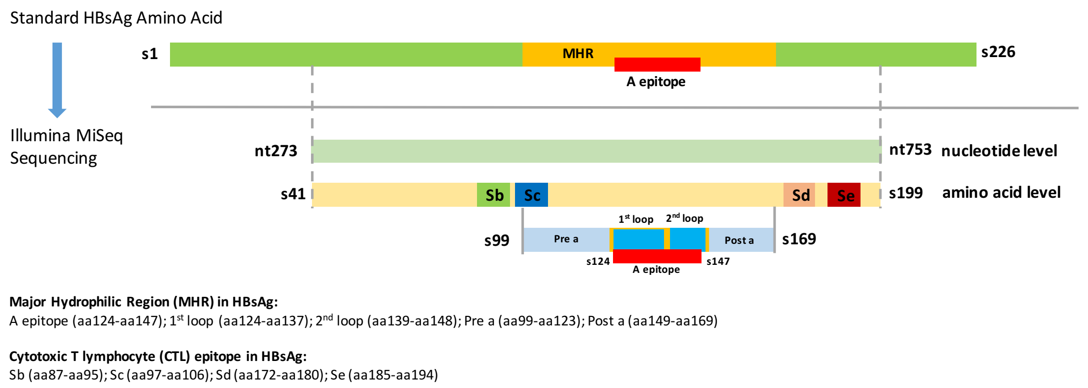

Figure S2. A schematic diagram indicating the studied fragment (s41-s169) in the whole HBV s region (s1-s226)

The HBV S region (s41-s199) was sequenced and segmented into two important regions: major hydrophilic region (MHR, s99-169) including Pre a (s99-123), 1st loop (s124-137), A epitope (s124-147), 2nd loop (s139-148) and Post a (s149-169), and cytotoxic T lymphocyte (CTL) epitopes including Sb (s87-95), Sc (s97-106), Sd (s172-180) and Se (s185-aa194). The position information of each region was based on the work of Prof. Lan-juan Li's team.

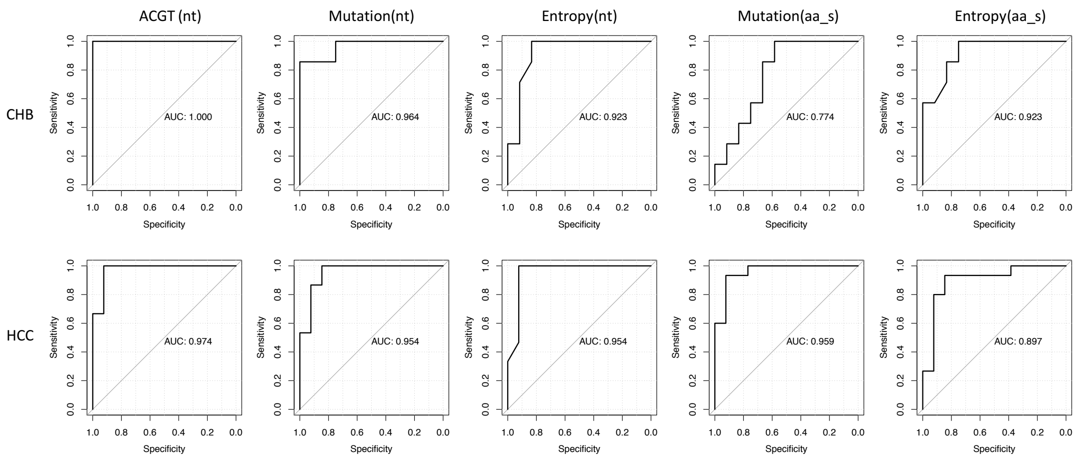

Figure S3. A schematic diagram indicating the studied fragment (s41-s169) in the whole HBV s region (s1-s226)

The HBV S region (s41-s199) was sequenced and segmented into two important regions: major hydrophilic region (MHR, s99-169) including Pre a (s99-123), 1st loop (s124-137), A epitope (s124-147), 2nd loop (s139-148) and Post a (s149-169), and cytotoxic T lymphocyte (CTL) epitopes including Sb (s87-95), Sc (s97-106), Sd (s172-180) and Se (s185-aa194). The position information of each region was based on the work of Prof. Lan-juan Li's team.

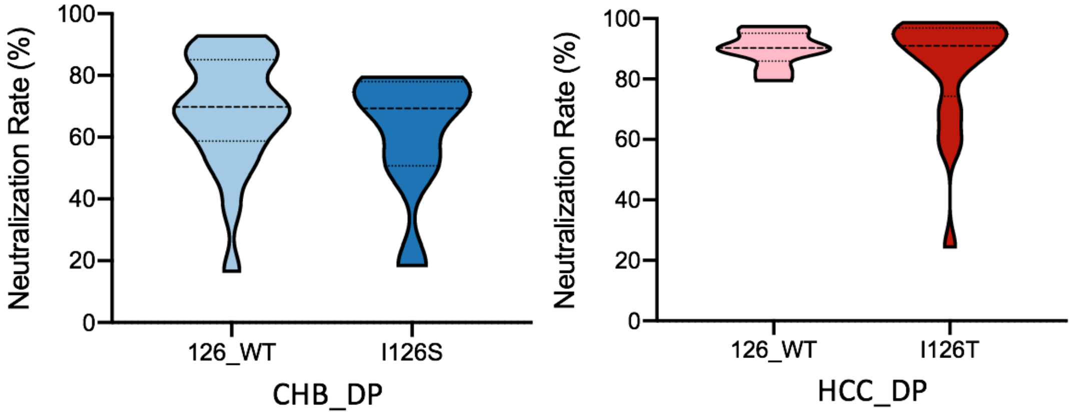

Figure S4. Comparison of the changes of neutralization rate between 126 wild-type and 126 mutations

Analysis of the change of neutralization rate proportion between 126 wild-type and I126S mutation in CHB_DP (left) or I126T mutation in HCC_DP (right) groups, respectively.

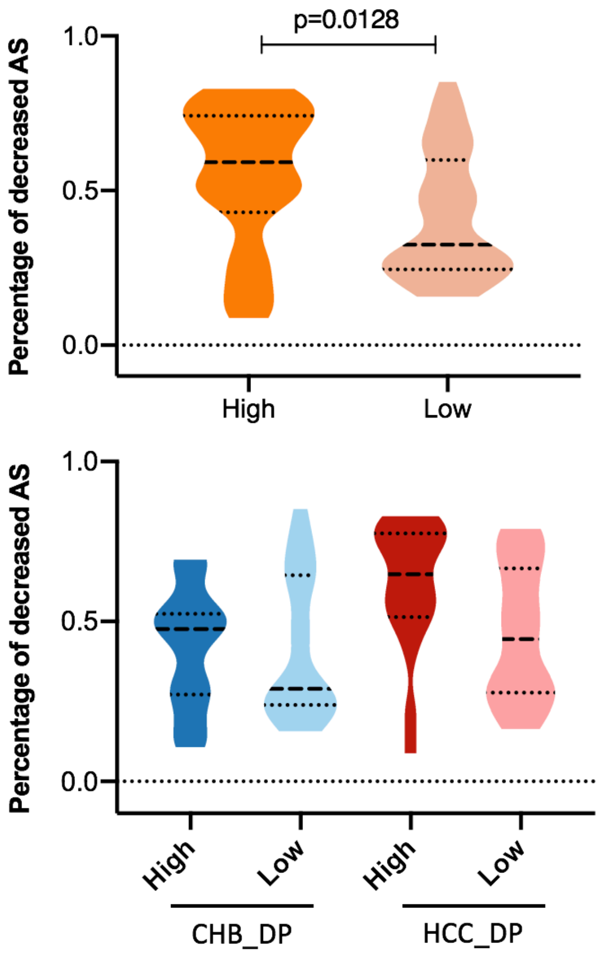

Figure S5. Comparison of the change of dp.AS between high and low accumulation of hotspot mutations groups in DP samples.

The violin plot demonstrated that decreased antigenicity score (AS) percentage in the MHR region of high accumulation of hotspot mutations groups in DP samples (dark-orange) was higher than that of low accumulation of hotspot mutations groups (light-orange), and the lower plot represented the difference of decreased AS percentage in four subgroups.

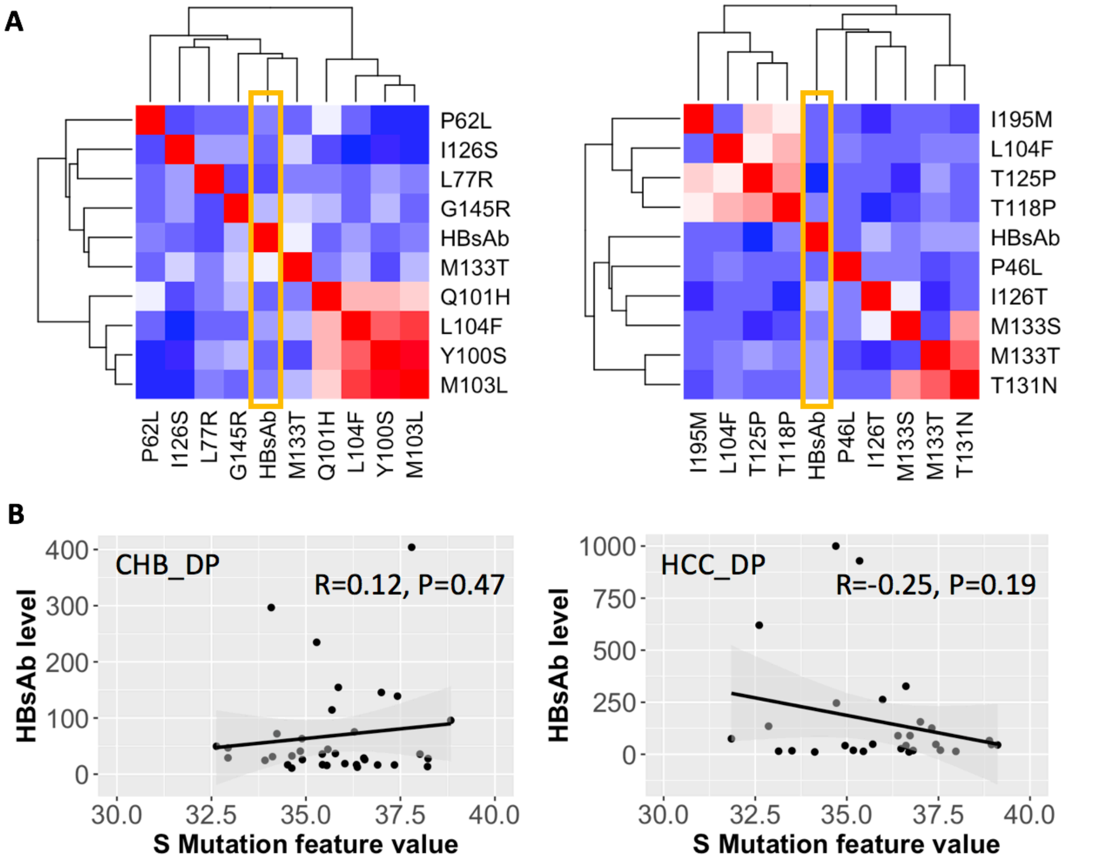

Figure S6. Correlation analysis of HBsAb level and HBV-S gene mutation in DP patients

A. Correlation analysis of HBsAb level and differential high-frequency mutations in CHB_DP (left) and HCC_DP (right) samples, respectively.

B. Correlation analysis of HBsAb level and the total mutation feature value of s gene in CHB_DP (left) and HCC_DP (right) samples, respectively.

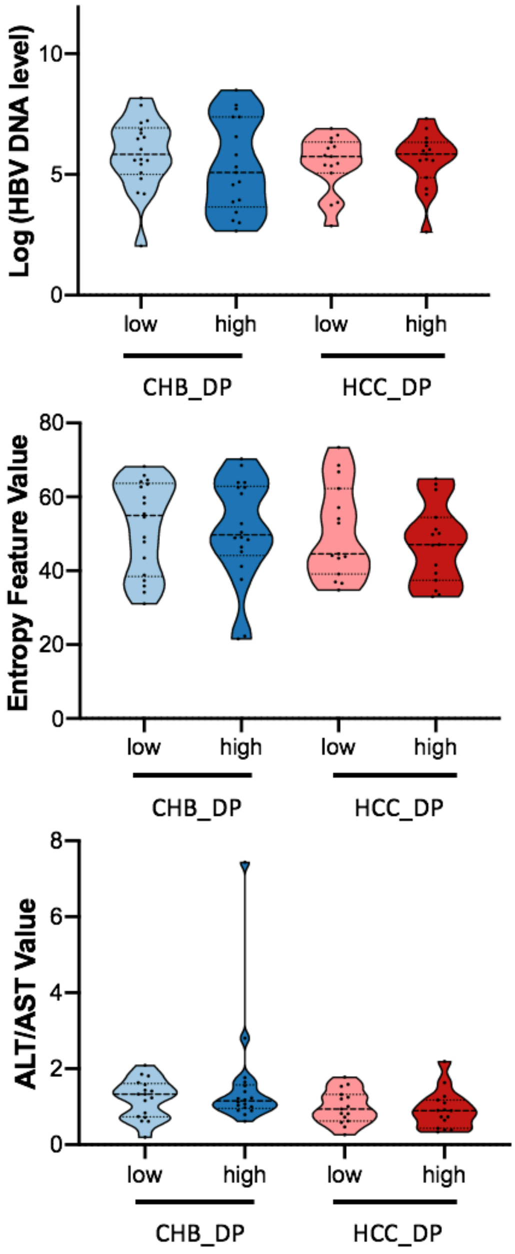

Figure S7. Comparison of HBV DNA, entropy feature and ALT/AST between high and low HBsAb groups

The violin plot showed the relationship of HBV DNA log (top), total entropy feature value (middle) and ratio of ALT to AST (bottom) between low and high HBsAb level groups in CHB_DP and HCC_DP, respectively.

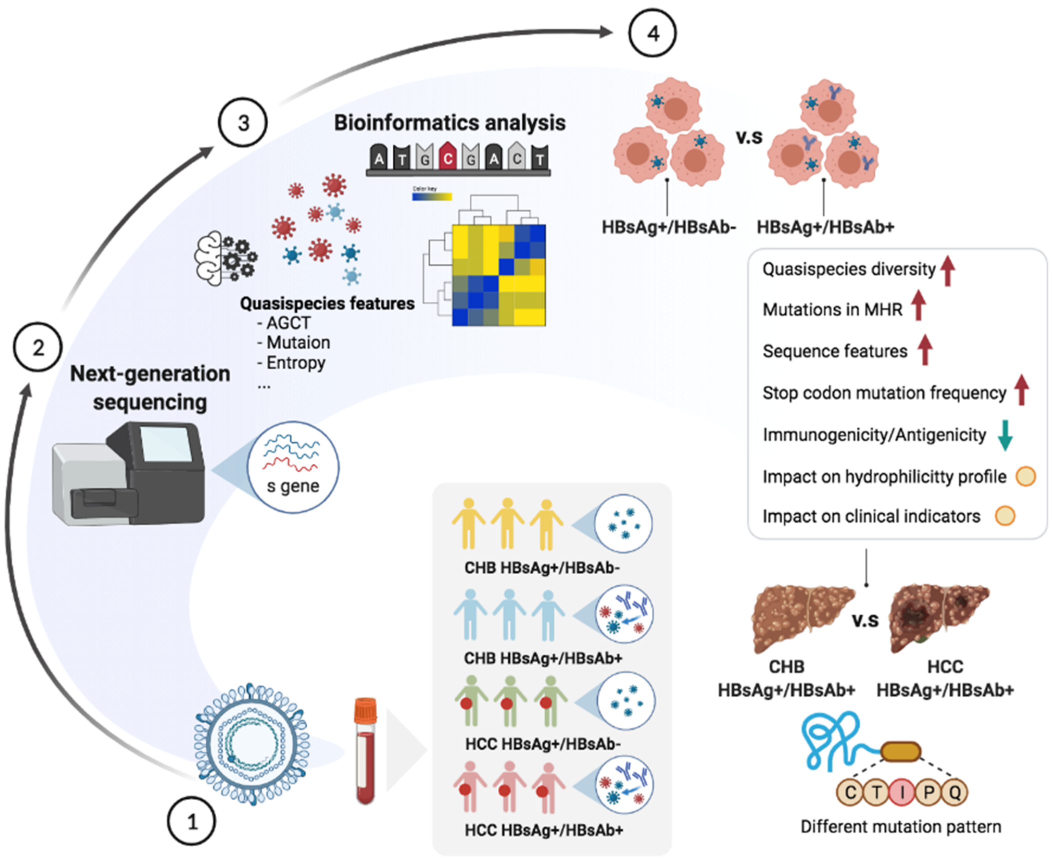

Graphical Abstract
